# PyNeon: A Python package for the analysis of Neon multimodal mobile eye-tracking data

**DOI:** 10.3758/s13428-026-03089-8

**Published:** 2026-06-29

**Authors:** Qian Chu, Jan-Gabriel Hartel, Alex Lepauvre, Lucia Melloni

**Affiliations:** 1 Neural Circuits, Consciousness, and Cognition Research Group, Max Planck Institute for Empirical Aesthetics, Frankfurt am Main, Germany; 2 Max Planck-University of Toronto Centre for Neural Science and Technology, Toronto, ON Canada; 3 Krembil Brain Institute and Institute of Biomedical Engineering, University of Toronto, Toronto, ON Canada; 4 Faculty of Psychology, Dresden University of Technology, Dresden, Germany; 5 Predictive Brain Department, Research Center One Health Ruhr, University Alliance Ruhr, Faculty of Psychology, Ruhr University Bochum, Bochum, Germany; 6 Department of Neurology, New York University Grossman School of Medicine, New York, NY USA

**Keywords:** Eye movements, Mobile eye-tracking, Pupil labs neon, Python, Open-source software, BIDS

## Abstract

Mobile eye-tracking has revolutionized the study of human behavior and cognition by enabling researchers to record eye movements in the real world. However, the dynamic and multimodal nature of mobile eye-tracking data also introduces significant analytical challenges, including the alignment, integration, and interpretation of complex data. To fill these gaps, we present PyNeon, a versatile, community-oriented Python package designed to streamline the analysis of mobile eye-tracking, motion, and video data from the Neon eye-tracking system (Pupil Labs GmbH). We describe how PyNeon provides accessible APIs for reading, preprocessing, epoching, and exporting Neon data. Furthermore, it supports advanced video processing such as mapping between eye movement data and real-world coordinates and dynamic scanpath estimation. PyNeon presents an open-source and extendable framework for analyzing mobile eye-tracking data and forms the foundation for higher-level applications.

**Supplementary Information:**

The online version contains supplementary material available at 10.3758/s13428-026-03089-8.

## Introduction

From the earliest investigations in classical experimental psychology – such as those by Helmholtz (1867), Yarbus (1967), and Stratton (1896) – eye movements have served as a window into the mind. These foundational studies revealed that visual perception is not a passive process. Instead, we use eye movements and fixations as our primary means to actively sample and explore the visual world (Holmqvist et al., 2011; Kowler, 2011; Martinez-Conde et al., 2004; Moca et al., 2011; Rolfs & Schweitzer, 2022).

Most contemporary research builds on this tradition, showing that eye movements are closely linked to a wide range of cognitive processes, including attention (Hoffman, 1998; Itti & Koch, 2000; Rizzolatti et al., 1987; van Ede et al., 2019), conscious perception (Frässle et al., 2014; Gollan & Raggam, 2025; Kronemer et al., 2022), memory (Hannula et al., 2010; Ryan & Shen, 2020; Voss et al., 2017), and decision-making (Orquin & Mueller Loose, 2013; Spering, 2022). Similarly, pupil size – once viewed narrowly as an index of arousal – has been increasingly recognized as a sensitive marker of cognitive effort, surprise, uncertainty, and other higher-order mental processes (Kahneman & Beatty, 1966; Grujic et al., 2024; Joshi & Gold, 2020; Schwiedrzik & Sudmann, 2020). Taken together, these lines of work reaffirm what classical studies first hinted at: the eyes are actively used to sample the visual world, opening a window to explore and characterize how perception and cognition unfold.

Most conventional eye-tracking experiments confine participants to tightly controlled, artificial settings, such as viewing stimuli on a static screen while maintaining head and/or body fixation. While these setups have been instrumental in advancing the field, studies presenting static stimuli are overly simplistic and ignore how behavior arises adaptively from the natural environment, limiting the generalizability of findings to real-world human behavior. Acknowledging these limitations, researchers have sought to bring eye-tracking research from the confines of artificial laboratory contexts to the real-world (Hayhoe & Ballard, 2005; Hayhoe & Rothkopf, 2011; Land, 2006; Nolte et al., 2026). This shift is fueled by the development of highly portable, head-wearable eye trackers, some of which are as unobtrusive as a pair of glasses, while maintaining accuracy to comparable degrees (Ehinger et al., 2019). These devices have opened new possibilities for the emerging field of neuroethology by enabling researchers to collect data in a much wider range of settings; studying eye movement patterns during locomotion (Matthis et al., 2018; Muller et al., 2024), social interactions (Hessels et al., 2022; Konovalova et al., 2021), artistic appreciation (Gulhan et al., 2024; Walker et al., 2017), among others.

Mobile eye-tracking also presents unique technical challenges. Unlike screen-based eye-tracking systems, mobile devices record gaze data relative to the wearer’s head position and orientation. Therefore, portable eye-tracking devices need to acquire additional contextual data – such as a video feed that captures the wearer’s view (i.e., a scene video) and motion data (from the head or the body) recorded by an inertial measurement unit (IMU) – to relate eye movements to the real world, which can be used, for example, to reconstruct the participant’s location in 3D space, or to help extract fixation events with higher precision. The richness of multimodal data, in turn, introduces challenges in data synchronization and integration. For instance, eye-tracking data typically has a higher sampling rate (e.g., 200 Hz) compared to the frame rate of the scene camera (e.g., 30 frames per second, fps). Resampling methods are thus desirable for data synchronization, especially in the face of potential data loss – a common problem with portable devices. The synchronization is essential for understanding oculomotor behavior in the real world with the aid of computer vision (CV) algorithms. Moreover, the naturalistic nature of experiments makes events of interest (e.g., viewing of a specific object) highly dynamic and variable, warranting novel ways of constructing event-based analyses.

To address challenges in eye-tracking data analysis, several open-source packages have been developed – among them GazeAlyze (Berger et al., 2012), eyetrackingR (Dink & Ferguson, 2015), Pymovements (Krakowczyk et al., 2023), and PupEyes (Zhang & Jonides, 2026). These tools have proven valuable for traditional, laboratory-based experiments that involve static screen stimuli with uniformly sampled gaze data acquired with stationary eye trackers. However, current open-source solutions remain limited when it comes to handling the full complexity of mobile eye-tracking. Specifically, none provides comprehensive tools for reading, synchronizing, and processing data beyond gaze and pupillometry (e.g., scene video or IMU), or for leveraging such multimodal data to address the unique challenges associated with dynamic, real-world environments. On the other hand, toolkits targeting specific challenges in mobile eye-tracking have recently emerged, including SocialEyes (Saxena et al., 2025), gazeMapper (Niehorster et al., 2025), and GazeClassify (Mueller & Mann, 2025). These packages are well suited to particular use cases (e.g., mapping gaze into world-centered reference frames or annotating scene video), but their specialization can make them less extensible for the diverse and rapidly evolving requirements of mobile eye-tracking research. Overall, the field still lacks a general framework for systematically managing and integrating multimodal mobile eye-tracking data that can serve as a backbone for advanced applications.

As a starting point toward addressing these challenges, we present PyNeon, an open-source package tailored to streamline the processing and analysis of multimodal eye-tracking data. Our package focuses on Neon, a modern head-worn eye-tracking system from Pupil Labs GmbH, and one of the many available devices for mobile eye-tracking. In this methods article, we introduce the initial release of PyNeon (https://github.com/ncc-brain/PyNeon) and provide an overview of its current core functionalities, including: data importation; basic signal processing and synchronization routines; data visualization; automated mapping of gaze data to scene videos; and export to standardized formats such as the Brain Imaging Data Structure (BIDS). Full details are available in the online documentation (https://ncc-brain.github.io/PyNeon/).

## Summary of the Neon system

The Neon system by Pupil Labs is a lightweight, wearable eye tracker engineered for real-world, mobile applications. Its modular design supports attachment to various head-worn frames, suitable for a wide range of natural behavior in dynamic, everyday settings.

**Fig. 1:**
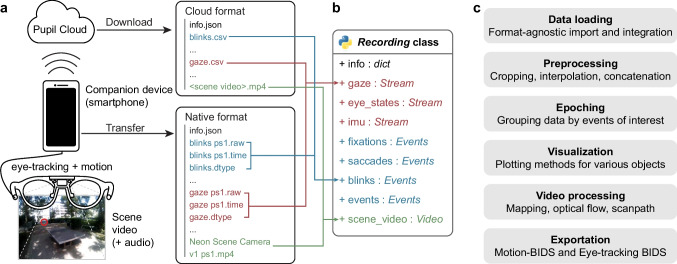
Overview of PyNeon. **a** *Left*: Diagram of Pupil Labs Neon mobile eye tracker with a typical data acquisition setup. The tennis table image illustrates a single frame from a scene video recorded during outdoor walking. The *red circle* indicates the concurrent gaze position. Data can be obtained either by direct extraction from the companion device or by download from Pupil Cloud. *Right*: Illustration of selected data files in Pupil Cloud or native format. **b** A Unified Modeling Language-like diagram that illustrates how files are imported into PyNeon instances and accessible as properties of the *Recording* class. *Arrows* between (a) and (b) denote the correspondence between exemplary source files and the *Recording* class properties. *Red* denotes data organized under the *Stream* class, *blue* denotes data organized under the *Events* class, and *green* denotes video-related data organized under the *Video* class. For full details on how PyNeon reads and organizes source files, refer to the online documentation. **c** Six main functionalities of PyNeon included in the first release

A detailed technical characterization of the Neon system has been provided elsewhere (Baumann & Dierkes, 2023); here, we briefly summarize its key components. The core module houses two high-speed eye cameras (nominally 200 fps) and an embedded real-time neural network for continuous gaze estimation. This system achieves a reported median accuracy < 1.5° with offset calibration (Baumann & Dierkes, 2023; Foucher et al., 2026). The module includes a nine-axis inertial measurement unit (IMU; TDK InvenSense Inc., San Jose, CA, USA; nominally 110 Hz) to capture head and body movements, supporting integrated analyses of gaze and locomotion. Visual input from the environment is recorded using a high-definition scene camera (103° × 77° field of view, 1600 × 1200 resolution, nominally 30 fps), allowing gaze data to be contextualized in a rich visual stream. When enabled, audio is recorded by dual microphones at a 44.1-kHz sampling rate and embedded into the scene video file. Data is streamed to a companion device, typically a smartphone that performs real-time processing and local storage (Fig. 1a, left).

Pupil Labs provides several tools for acquiring and analyzing data collected with the Neon eye-tracking system. During data acquisition, a companion device records data in a proprietary native format (Fig. 1a, bottom right). These native data can be accessed programmatically using the Pupil Labs Neon Recording Python library (https://github.com/pupil-labs/pl-neon-recording/) or explored visually using the interactive desktop application Neon Player (https://docs.pupil-labs.com/neon/neon-player/). In addition, native recordings can be uploaded to Pupil Cloud (https://cloud.pupil-labs.com/), where a standardized processing pipeline is automatically applied. This pipeline performs preprocessing and organization of the data into a structured hierarchical format that can be downloaded for local use (see official documentation at https://docs.pupil-labs.com/neon/data-collection/data-format/; Fig. 1a, top right). Pupil Cloud further supports post hoc analyses, including data visualization and gaze mapping onto surfaces using reference images or marker-based systems. Notably, multiple key features of Pupil Cloud – including unlimited storage, anonymization, and full access to analysis algorithms – require a paid subscription, which limits its accessibility to users.

Inspired by these tools, PyNeon is an open-source software package that integrates core functionalities of the official Pupil Labs ecosystem while extending them with features tailored to research workflows. PyNeon can read data stored in either the native format or the Pupil Cloud-processed format through a unified application programming interface (API), internally harmonizing both formats (Fig. 1b). For example, column names from native recordings are automatically remapped to the more descriptive and standardized Pupil Cloud naming scheme (e.g., the ambiguous “x” to “gaze x [px]”). By supporting both data formats through a single API, PyNeon serves as a central hub for analysis, ensuring that workflows remain consistent and transferable regardless of the source format. Beyond data loading, PyNeon offers an end-to-end analysis framework that includes preprocessing, epoching, surface mapping, and dynamic scanpath estimation. This integrated approach enables efficient quantitative processing, visualization, and management of eye-tracking data throughout the analysis process. The following sections describe each functionality in detail.

## Importing hierarchical Neon data

PyNeon provides an input/output class, *Recording*, to effortlessly import/export various types of data associated with a single recording session. A *Recording* object is initialized by specifying the path to the recording directory. This object tracks the files present in the directory based on the naming conventions from Pupil Labs. Data is read into computer memory only when the corresponding property, such as *Recording.gaze* or *Recording.scene_video*, is accessed (Fig. 1b). This design allows users to quickly access each data modality and flexibly accommodates recordings with an incomplete set of files, for example, when recordings are downloaded without the accompanying scene video.

PyNeon reads metadata from JSON files (e.g., info.json) into Python dictionaries. The scene video in MP4 format, along with its timestamps and metadata, is read into a *Video* object, which contains an OpenCV *VideoCapture* object (Bradski, 2000). In the current release, PyNeon does not natively process audio data which is optionally embedded in the scene video; however, users can manually extract the audio track from the.MP4 file for custom processing. Tabular timeseries or events data – stored as CSV files when downloaded from Pupil Cloud, or as binary files in the native format – are read into Pandas *DataFrame* objects (McKinney, 2010) and attached to either an instance of *Stream* or *Events*.

**Fig. 2:**
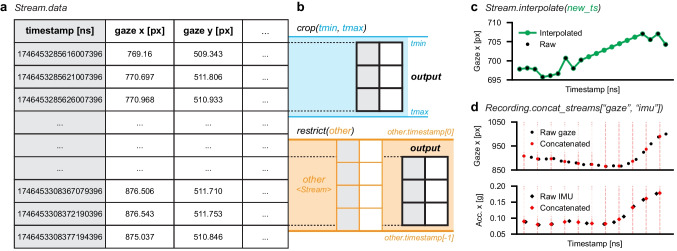
Core data structure and preprocessing functionalities in PyNeon. **a** Example of a tabular data structure underlying a PyNeon *Stream* object. Data are stored as a Pandas *DataFrame* (e.g., loaded from gaze.csv), where each row corresponds to a sample and the index represents UTC timestamp in nanoseconds (“timestamp [ns”]). Columns contain measurements such as gaze coordinates and other stream-specific variables. **b** Basic preprocessing methods of the *Stream* class: The *Stream.crop(tmin, tmax)* method extracts a time window from a stream. The *Stream.restrict(other)* method aligns two streams by trimming one stream to the temporal range of another, facilitating subsequent multi-stream analysis. **c** The *Stream.interpolate()* method enables resampling of data to a custom set of timestamps – for example, to enforce uniform sampling. Interpolated values at new timestamps are shown in *green*, original samples in *black*. **d** The *Recording.concat_stream()* method allows for the temporal alignment of multiple data streams by interpolating them to the same set of timestamps. After concatenation, new data points (*red*) are aligned to a common and uniform temporal grid (*red dashed lines*)

A *Stream* object contains time-series data, including gaze, eye states, and IMU. *Stream* data is acquired in a semi-continuous manner, with a UTC timestamp with nanosecond precision, indexing each data point with multiple measurements (example shown in Fig. 2a). In contrast, an *Events* object contains discrete events indexed by timestamps. These events can be physiological, including blinks, saccades, and fixations detected by Neon (Dierkes et al., 2018; Drews & Dierkes, 2024); or user-supplied, for instance, triggers sent to Neon during the recording, or event annotations made post-hoc. *Events.data* includes the start and end timestamps, durations, and event descriptions. Beyond data output by Neon, any tabular data indexed by the same timestamp format can be used to initialize *Stream* or *Events* objects. This allows users to process custom data, including manually or automatically annotated events (e.g., audio-derived stimulus onset), or streams from additional sensors with the full functionality provided by these classes.

By leveraging Pandas and OpenCV to manage tabular and video data, PyNeon offers users access to whole suites of functionality from these libraries. Pandas is a versatile library that efficiently manipulates and analyzes tabular data through powerful data structures and diverse operations. OpenCV can efficiently process images and videos and provides native integration with computer vision methods. Additionally, the specialized *Stream*, *Events*, and *Video* classes include tailored attributes and methods for common operations and integrative analyses. For example, simply calling *Stream.duration* allows users to retrieve the stream’s duration in seconds, dynamically computed from the associated data; Or, users can correct the fisheye camera distortion in scene video by using *Video.undistort_video()* to generate a new scene video with the distortion corrected. In the following sections of the manuscript, we will provide an overview of additional methods that are crucial for mobile eye-tracking data analysis.

## Preprocessing of data streams

PyNeon provides a comprehensive suite of preprocessing tools designed to address specific challenges encountered with mobile eye-tracking data. Its functionalities enable users to refine, synchronize, and align multimodal data streams, offering methods for data cropping, flexible interpolation, concatenation, and robust synchronization of gaze with video frames.

One preprocessing step is data cropping, useful when extracting data within a specified time window or aligning the start and end times of different data streams. PyNeon *Stream* objects thus come with a *crop()* method that allows cropping based on user-defined start and end times in various time formats (UTC nanoseconds/relative time/sample number) (Fig. 2b, upper). Moreover, the *Stream.restrict()* method limits one stream data to the timespan of another by cropping to the latter’s first and last timestamps (Fig. 2b, lower).

A key challenge of portable eye-tracking devices is their lightweight design, which limits their computational power compared to larger systems like laptops. Consequently, hardware limitations may lead to *Stream* data losses or temporal gaps between data points. If data were assumed to be regularly sampled at the nominal sampling rate, disregarding the actual timestamps, such irregular sampling can accumulate into substantial temporal asynchrony. To address this, PyNeon offers a *Stream.interpolate()* method that leverages the *interp1d* class from SciPy (Virtanen et al., 2020) to interpolate *Stream* data to a custom set of (uniformly spaced) timestamps. An example is shown in Fig. 2c, where lost samples are visibly “filled in”. By default, *Stream.interpolate()* performs linear interpolation for columns of stream data of float type, and nearest interpolation for columns of other data types. To avoid unreliable interpolation across large temporal gaps, PyNeon emits warnings and returns missing values when gaps exceed a configurable threshold (*max_gap_ms*).

This interpolation method, in turn, lays the foundation for other utilities. For example, the *Stream.interpolate_events()* method allows users to interpolate data points surrounding physiological events (e.g., interpolating pupil size around blinks, during which the pupils are invisible to the camera and data should therefore be considered invalid). Additionally, the *Recording*-level method *concat_stream()* enables concatenating multiple streams. The method interpolates selected streams onto a shared set of timestamps (with user-configurable sampling rate), so that data originally sampled at different frequencies (e.g., 200-Hz gaze and 110-Hz IMU) is fully synchronized after concatenation (Fig. 2d).

Moreover, since Neon gaze data is reported in pixel coordinates of the scene camera, it often needs to be interpreted in the visual context captured by the scene camera. However, the scene video has a much lower frame rate (30 fps) than gaze data (200 Hz). This disparity makes downsampling the gaze stream to match the video frames essential for studies requiring precise integration of gaze measurements with scene video frame-derived information – for example, rapid serial visual presentation experiments. While interpolating gaze to video timestamps could be one solution, it is prone to transient noise around each video frame, as only two nearby data points are used for estimation. Therefore, PyNeon instead recommends the *Recording*-level method sync_gaze_to_video(), which uses a rolling window average (*Stream.window_average()*) to estimate the mean gaze location at the time a video frame is acquired. Compared to interpolation, this approach better reflects the continuous evolution of naturalistic scenes and offers greater robustness to signal fluctuations. It is important to note that applying a window average alters the frequency characteristics of the gaze signal, and thus should be used with caution when spectral properties are of interest. In later sections, we will show how to use the synchronized gaze data in conjunction with the scene video to enable advanced gaze-video mapping.

## Event-based analyses via epoching

A fundamental step in time series analysis is segmenting data into time windows around events of interest, i.e., epochs. In mobile eye-tracking, such events could be the presentation of particular stimuli (e.g., faces), the participant viewing certain scenes, or reaching specific locations. Through epoching, researchers can easily gain insights on whether oculomotor patterns occur systematically around predefined events (for example, see Nolte et al. (2025)).

PyNeon offers the *Epochs* class to flexibly construct, visualize, and analyze epochs. *An Epochs* object is initialized by supplying a source data object (*Stream* or *Events*) and a *DataFrame* containing time information about the epochs. Each row of the *DataFrame* contains the reference time (e.g., stimulus onset time), the time window (how much time before and after the reference time to extract data from), and a description string (to differentiate epochs of different types, e.g., stimulus 1 versus stimulus 2). The time information *DataFrame* can be defined manually or automatically extracted from an *Events* object with the helper function *events_to_epoch_info()*.

**Fig. 3:**
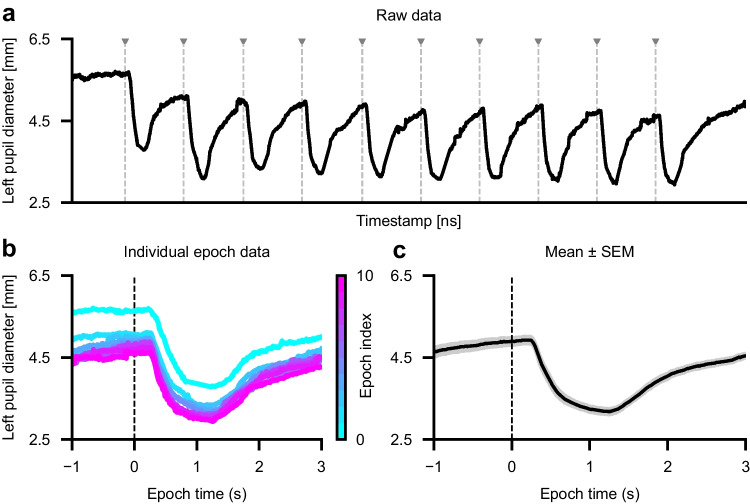
Pupillary light reflex analyzed through epoching. **a** Raw time series of left pupil diameter recorded during an experimental display. *Grey dotted lines* indicate the times of black-to-white screen switches; A transient pupil constriction is visible following each. See text for details of the paradigm. The *x*-axis reflects the original Neon timestamp format (UTC timestamp with nanosecond precision). **b** Data epoched around switches. Each colored trace represents a single epoch extracted from the same underlying recording shown in panel (a), aligned to the event time (0 s). **c** Average pupil diameter across epochs (mean ± SEM). The mean response illustrates the characteristic pupillary constriction following luminance increases. *SEM* standard error of the mean

Figure 3 illustrates a representative epoching analysis pipeline using a sample recording. The participant fixated on a monitor screen that alternated between black and white (duration = 750 ms) every 3 s in a dark room. Concurrent with each luminance switch, an event was sent to Neon via the real-time network API. This paradigm was designed to trigger the well-documented pupillary light reflex (Ellis, 1981), which benchmarks the validity of the analysis. Figure 3a displays the left pupil diameter throughout the recording, with grey dotted lines marking each luminance switch. While it is observable that the pupil narrows following each switch, epoching provides a clearer view.

To construct epochs, we extracted the timestamps of the switch onsets from *Recording.events* (which reads data from events.csv) as the reference time. We set epoching time windows to span from – 1 to 3 s relative to the reference time. Since only one event type was considered, the event type (“switch onset”) itself served as the description. The *Epochs.plot()* method generated the trial-by-trial epoched pupil traces depicted in Fig. 3b.

Note that in the example, the source data was a *Stream* of eye states, with a consistent time window (– 1 to 3 s) applied to all epochs. Under such conditions, the epoch data can also be converted to a three-dimensional NumPy array (Harris et al., 2020) with shape (${n}_{\mathrm{e}\mathrm{p}\mathrm{o}\mathrm{c}\mathrm{h}\mathrm{s}}\times {n}_{\mathrm{m}\mathrm{e}\mathrm{a}\mathrm{s}\mathrm{u}\mathrm{r}\mathrm{e}\mathrm{m}\mathrm{e}\mathrm{n}\mathrm{t}}\times {n}_{\mathrm{t}\mathrm{i}\mathrm{m}\mathrm{e}\mathrm{s}}$) by calling the *to_numpy()* method. Internally, this is achieved by interpolating epoched data to a common temporal grid relative to the epoch reference time. The 3D array format allows users to perform efficient numerical operations and effortlessly compute various statistical measures. For example, in Fig. 3c, we plotted the mean and standard error of the mean of the converted 3D data. Taken together with the trial-wise plot, these analyses show that pupil constriction consistently occurred approximately 300 ms after the screen switched in luminance.

**Fig. 4:**
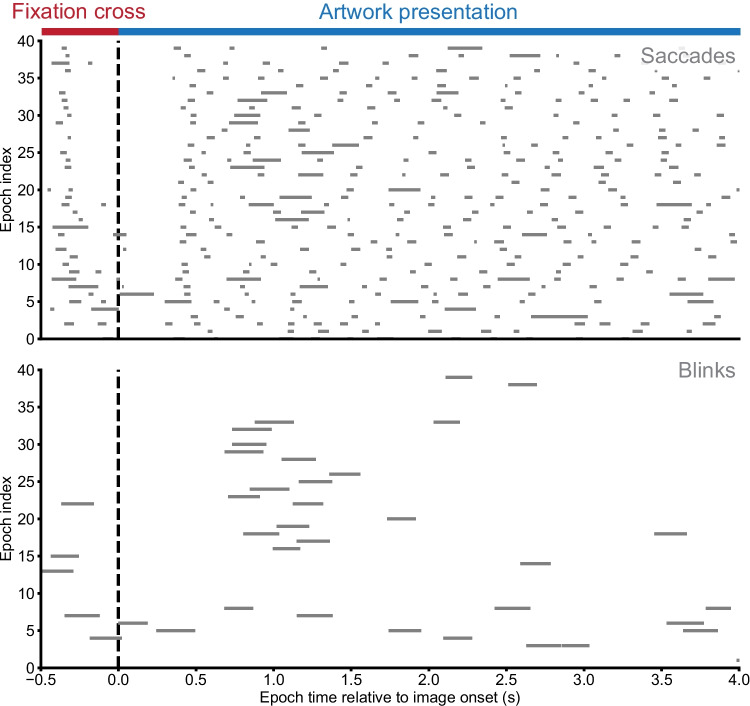
Epoching saccades and blinks during viewing of artworks. Raster plots showing the epoched saccades (*top*) and blinks (*bottom*) across 40 epochs, aligned to artwork presentation onset (*vertical dashed line* at 0). Each *short gray line* represents a single event, with line length indicating saccade or blink duration. Individual epochs are arranged along the *y*-axis. The *x*-axis shows time relative to artwork onset. The periods corresponding to the fixation cross (*crimson*) and artwork presentation (*cyan*) are indicated above the plots

In addition to the earlier example using a *Stream* as the data source, *Epochs* can also be constructed from an *Events* object. To illustrate this, we show results from an experiment where a participant freely viewed 40 artworks, each presented for 4 s following a 500-ms central fixation cross. We epoched saccade and blink events from – 0.5 to 4 s relative to artwork onset and visualized trial-by-trial event durations using *Epochs.plot()* (Fig. 4). These plots reveal that saccade frequency increased markedly around 480 ms after stimulus onset, reflecting a shift from rapid, bottom-up scene gist perception to active exploration. In contrast, blinks were relatively rarer and less consistent in time relative to the onset of artworks.

## Surface mapping using fiducial markers

In many experiments, researchers aim to understand gaze behavior on surfaces such as computer screens, artworks, or projections. Traditional eye-tracking systems typically handle this by performing a calibration procedure at the beginning of the experiment, where participants are asked to fixate on a series of known points on the surface. This allows the system to compute a fixed transformation that maps the raw gaze data (usually in eye-centered coordinates) to the coordinates of the surface, such as a screen. Once established, this mapping remains stable because both the participant’s head and the surface remain relatively fixed throughout the recording. However, in mobile eye-tracking, this transformation cannot remain static. The participant – and therefore the eye tracker – moves through the environment, and the relevant surfaces may change or shift in the field of view. As a result, the gaze-to-surface mapping must be dynamically updated over time. This introduces a major challenge: how to continuously and accurately estimate where someone is looking in relation to changing reference frames. Addressing this issue is key to extending traditional gaze analysis techniques (like fixations, scanpaths, and heatmaps) into more naturalistic, mobile contexts.

Fiducial markers such as AprilTags (Olson, 2011) and ArUco (Garrido-Jurado et al., 2014) offer a robust and lightweight solution for establishing spatial references in dynamic environments. These high-contrast visual markers can be unobtrusively placed on or around the target surface of interest – such as a screen, poster, or physical object. Because their positions in the physical world are known, they act as stable spatial anchors. When captured in video frames from a mobile eye tracker, their detection enables the computation of precise transformations between the video and the tagged surface. This allows gaze data to be mapped to the relevant surface even as the participant or the environment moves.

**Fig. 5:**
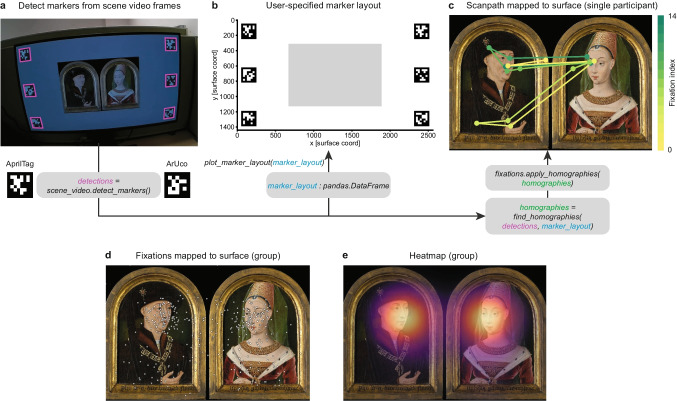
Mapping eye movements to a surface using fiducial markers. **a** Exemplary scene video frame showing six AprilTags displayed near the borders of a computer screen. Magenta lines connect the four corners of each marker detected by the *detect_markers()* method. **b** Visualization of the user-supplied marker layout obtained by the *plot_marker_layout()* function, representing ground truth knowledge about the locations of markers in the surface coordinates. **c** Frame-wise homographies are estimated using marker detections and marker layout, which can be applied to gaze or fixation data to obtain the gaze location in surface coordinates. The top image illustrates an exemplary scanpath of a single participant mapped to the artwork. The color gradient denotes temporal sequence of fixations. **d** Fixations of 35 participants mapped onto the reconstructed screen frame. **e** Fixation heatmap obtained by smoothing the group fixation points with a Gaussian kernel. The artwork used in the example recording is under Creative Commons Zero Public Domain Designation and is reproduced from: Flemish. *Philip the Good, Duke of Burgundy; Isabelle of Bourbon*, 1510–1530. The Art Institute of Chicago

PyNeon provides a flexible and modular pipeline for integrating fiducial markers into surface-based gaze and fixation analysis. The *Video.detect_markers()* method identifies markers within each frame of the scene video and returns their unique marker ID along with the coordinates of their corners (Fig. 5a). When the physical positions of these markers (*marker_layout*, Fig. 5b*)* are supplied in the surface coordinate system (e.g., screen pixel space), PyNeon’s *find_homographies()* method can estimate a frame-wise transformation matrix between camera and surface coordinates, known as a homography. These homographies are then used by the *apply_homographies()* method of a gaze or fixation *Stream/Events* instance to map gaze and fixation data from scene camera coordinates onto surface coordinates. Further details, including customizable parameters and full workflow, can be found in Supplementary Information. This flexibility highlights an advantage of PyNeon over Pupil Cloud, which also offers AprilTag-based mapping but through a fixed workflow in which marker detection parameters and output formats are not user-configurable. Note also that unlimited access to this functionality on Pupil Cloud requires a paid subscription.

In Fig. 5c–e, we show the results for an experiment (similar to the one described in Fig. 4) where participants (*N* = 35) viewed artworks on a computer screen. Six AprilTags (tag family = 36h11) were displayed in the corners of the screen and detected by *Video.detect_markers()*. Successfully detected markers can be visualized on top of the scene video frame by calling *Video.plot_detections(),* as showcased in Fig. 5a. To establish a correspondence between the camera coordinates of the detected markers and the ground truth placement of the markers (e.g., at which locations of the screen were the markers actually presented, in pixels), users need to specify the marker layout, which can be visually verified by the *plot_marker_layout()* function as in Fig. 5b. Figure 5c illustrates the sequence of mapped fixations from one participant viewing the image, also known as a scanpath. At the group-level, we can probe gaze behavior across participants by plotting the fixations on the surface in Fig. 5d and the corresponding gaze density map in Fig. 5e.

For situations where placing physical markers on a surface is impractical or undesirable, PyNeon provides an alternative mapping method that detects a rectangular contour – such as the edge of a bright screen – based on luminance contrast (Supplementary Fig. 1). Since contours are more susceptible to occlusion at corners and more prone to false detections than fiducial markers, manual fine‑tuning of detection parameters may be required to achieve optimal performance. Further details about this method are provided in the Supplementary Information.

## Dynamic scanpath estimation with optical flow

Beyond the information provided by fixation density – that is, where subjects tend to look most often – scanpaths, or sequences of fixations, offer a rich and complementary source of insight into cognitive processes. For example, the act of revisiting previously viewed locations serves as a critical marker of memory recollection, even in the absence of verbal reports (Kragel & Voss, 2022; Nikolaev et al., 2024; Ryan et al., 2000). Additionally, the fixation sequence plays a central role in modern models of visual saliency and gaze prediction (Kümmerer & Bethge, 2023).

While the estimation of scanpaths is straightforward when a well-defined and, ideally, static reference frame is available as in Fig. 5e, it becomes markedly more challenging in truly mobile settings. After all, the real world is dynamic: people walk past, cars pass by, and clouds drift overhead. From the perspective of a camera, the coordinates of the same object constantly change. Consequently, we cannot assume that the location of a past fixation still corresponds to the originally fixated object. To obtain accurate estimates of past fixations, we need to actively account for the evolving visual scene and the movement of objects within it.

In computer vision, this challenge of apparent motion of objects across a sequence of video frames is known as optical flow. One effective approach to address it is tracking how small image patches surrounding fixation points move from frame to frame (Lucas & Kanade, 1981), thereby estimating apparent motion regardless of whether that motion arises from observer movement, object movement, or both. PyNeon implements this technique using the pyramidal Lucas-Kanade (LK) optical flow algorithm from OpenCV (Bouguet, 1999; Bradski, 2000), with additional implementation details provided in the Supplementary Information. This algorithm estimates motion in a hierarchical, coarse-to-fine manner, enabling it to robustly track both large and small displacements across frames.

**Fig. 6:**
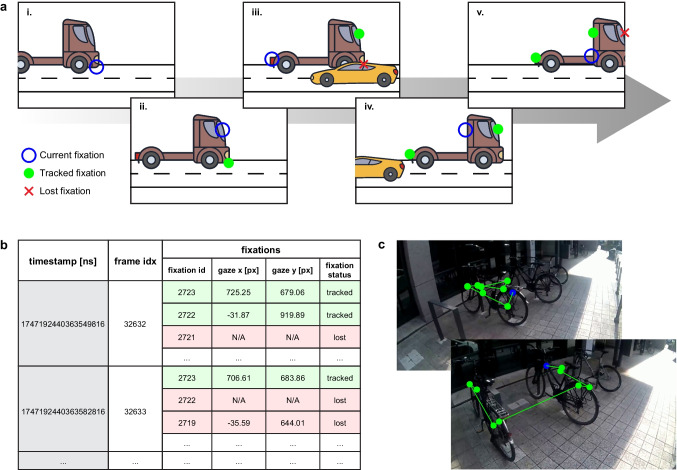
Dynamic scanpath estimation. **a** Sequence of frames showcasing the principles of scanpath estimation in dynamic environments. i.) Ongoing fixation (*blue*) on the front of the truck. ii.) As a new fixation occurs, the past fixation gets passed to the optical flow algorithm and tracked (*green*). iii.) As the yellow car occludes the front of the truck, the previously tracked fixation is lost (*red*) because the image features used for tracking are no longer visible. iv.) Lost fixations are no longer tracked as the algorithm has no global model or memory of the scene. (v.) Once a fixation travels past the edge of the video frame, it also becomes lost. **b** Scanpath data format. Scanpath data is saved as a nested *DataFrame* indexed by video timestamps. Each row contains the corresponding frame index and a sub-*DataFrame* containing information about all ongoing, tracked, and newly lost fixations (including fixation coordinates and tracking status). **c** Two nearby frames with scanpath overlaid in a real outdoor recording, demonstrating how tracked fixations remain aligned with the underlying scene content across frames

The result is a dynamic scanpath that remains aligned with the evolving visual content. Each fixation is annotated with a status flag that indicates whether it is ongoing, successfully tracked, or lost – e.g., due to occlusion, tracking drift, or moving out of frame – as illustrated in Fig. 6a. Users can adjust optical flow parameters to optimize performance in challenging scenarios, such as poor lighting or low-contrast surfaces. The resulting scanpath is returned as a frame-synchronized *Stream* object and can be visualized using *overlay_scanpath_on_video()*, as shown in Fig. 6b and c.

## Exporting data to Brain Imaging Data Structure (BIDS) formats

Adopting standardized data formats is essential for ensuring transparency, reproducibility, interoperability, and data sharing in research. The Brain Imaging Data Structure (BIDS) provides a widely accepted framework for organizing and sharing behavioral and neuroimaging data in a consistent, machine-readable way. Originally developed for magnetic resonance imaging datasets (Gorgolewski et al., 2016), BIDS has since been extended to cover a variety of data modalities (Poldrack et al., 2024). Two BIDS extensions are particularly relevant for mobile eye-tracking: Motion-BIDS (BEP029), which standardizes motion data including acceleration and angular velocity from IMUs (Jeung et al., 2024); and Eye-Tracking-BIDS (BEP020), which organizes gaze position and pupil data (Szinte et al., 2026). Together, these extensions capture participants’ locomotion and head movements as well as eye-movement behavior.

Preparing recordings to conform to BIDS is, however, non-trivial. BIDS prescribes specific file formats (e.g., TSV/TSV.GZ) and detailed accompanying metadata (e.g., JSON sidecars), as well as dataset-level files and a standardized directory hierarchy. Building conversion pipelines can be challenging for beginners, particularly when working with data from newer devices such as Neon. For example, the existing eye2bids conversion tool currently supports conversion only from EyeLink systems to Eye-Tracking-BIDS (Szinte et al., 2026), limiting broader adoption of BIDS for mobile eye-tracking datasets.

**Fig. 7:**
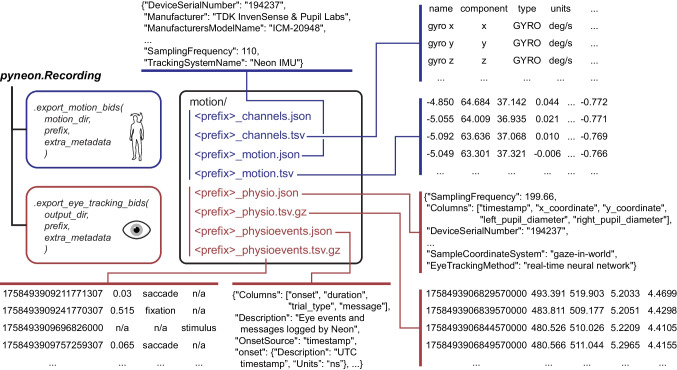
Exporting data to Motion-BIDS and Eye-Tracking-BIDS. Workflow and output files of PyNeon’s exporting to BIDS functionalities. *Blue* and *red* denote workflows related to exportation to Motion-BIDS and Eye-Tracking-BIDS, respectively. The human silhouette and eye drawings are adapted from Zane Mitrevica and John Chilton’s contributions to Scidraw.io (Chilton, 2020; Mitrevica, 2021) and are licensed under Creative Commons 4.0 (CC-BY)

PyNeon provides convenience methods for exporting Neon recordings into BIDS-compatible files (Fig. 7). For Motion-BIDS export, users can call the *Recording*-level method *export_motion_bids()*, specifying an output directory, a filename prefix (typically containing the relevant BIDS entities, e.g., sub-, ses-, task-, run-), and optionally additional metadata. The method then automatically generates the four Motion-BIDS files (<prefix>_motion.tsv, <prefix>_motion.json, <prefix>_channels.tsv, <prefix>_channels.json). Device-specific metadata is extracted from the recording’s info field (e.g., serial number) and combined with relevant technical specifications from the associated Neon module. Additionally, users can pass a Python dictionary to include custom metadata fields (e.g., task name, instruction, institution name). These entries are automatically incorporated and updated in the corresponding JSON files, allowing for flexible annotation while preserving standardization.

Similarly, for Eye-Tracking-BIDS export, users can call *Recording.export_eye_tracking_bids()* with an output directory, and optionally a filename prefix and extra metadata. The resulting <prefix>_physio.tsv.gz contains timestamped gaze position and pupil diameter measurements, while events such as fixations, saccades, blinks, and logged messages are stored in <prefix>_physioevents.tsv.gz. Corresponding JSON sidecars are generated automatically and can be enriched with user-supplied metadata.

With only a small amount of additional metadata (e.g., dataset_description.json) and appropriate organization of outputs within the BIDS directory structure, PyNeon-exported datasets can be made fully BIDS-compliant and have passed validation checks using the BIDS Validator (RRID:SCR_017255, https://github.com/bids-standard/bids-validator). By supporting export to BIDS, PyNeon helps users align mobile eye-tracking data with the growing ecosystem of standardized behavioral and neural datasets, facilitating integration with existing analysis pipelines, enabling easier sharing with collaborators or the community, and lowering barriers to future reanalysis and reuse.

## Discussion

Mobile eye-tracking holds the promise of transforming research on human cognition and behavior by transcending the constraints of traditional laboratory settings. Mobile eye-tracking allows participants to navigate – not on screens or in virtual worlds, but in their real, lived environment; to appreciate art – not on computer screens, but in galleries, museums, and concert halls; to interact socially – not with fictional characters, but real humans: Strangers on the streets, students in classrooms, family members at home. As such, it contributes to the ongoing push for human neuroethology – understanding human behavior and cognition, not in the narrow confines of laboratories, but in the real world. While mobile eye-tracking has already enabled many naturalistic behavioral studies (Hessels et al., 2022; Muller et al., 2024; Nolte et al., 2025; Walker et al., 2017), analyzing such datasets remains technically demanding due to the complexity of multimodal recordings and dynamic visual environments.

However, conducting mobile eye-tracking research in dynamic environments presents substantial technological and analytical challenges. Data from diverse sensors – such as scene cameras, inertial measurement units, and gaze trackers – are acquired in distinct formats, with differing sampling rates and vulnerability to unique sources of noise or data loss. Crucially, these streams cannot yield insight individually, but must be synchronized and integrated to create a coherent picture of the participant’s behavior and context. Existing software, designed for static, screen-based setups, is ill-equipped to handle this complex, multimodal interplay.

PyNeon was developed specifically to address these needs. Rather than introducing fundamentally new algorithms, PyNeon focuses on integrating established computational methods into a coherent workflow tailored to mobile eye-tracking datasets. It draws inspiration from time-series processing software like MNE (Gramfort et al., 2013) and Pynapple (Viejo et al., 2023), positioning itself as a flexible backbone for mobile eye-tracking research using Neon. By providing data structures across a rich collection of modalities (*Video, Stream, Events, Epochs*), PyNeon simplifies the handling of multimodal data. By designing a wide range of preprocessing methods, PyNeon facilitates efficient data manipulation, quality control, and integration. Advanced computer vision-based methods, including surface mapping and dynamic scanpath estimation, further accelerate the process of turning complex and dynamic data into scientific insight. Taken together, the comprehensive suite of methods streamlines data handling and analysis. This allows researchers to focus on what they do best: asking questions, designing experiments, and interpreting results.

### A community built by and for researchers

PyNeon was built with a deep commitment to open science and collaborative development. While it is designed for seamless integration with Neon data, it is an independent, community-driven initiative unaffiliated with Pupil Labs. This approach ensures that the toolbox remains open, collaborative, and adaptable to the evolving needs of the research community. PyNeon is designed to complement rather than replace existing Python scientific libraries, building on widely used packages such as NumPy, Pandas, SciPy, and OpenCV. The codebase is fully open source under the MIT license, supporting free and unrestricted use. PyNeon is distributed via the Python Package Index (https://pypi.org/project/pyneon/), facilitating installation and adoption across a broad range of research environments. Addressing the reproducibility crisis and advancing open science, PyNeon embodies the FAIR (Findable, Accessible, Interoperable and Reusable) principles (Wilkinson et al., 2016; Zheng et al., 2024) by building on open-source libraries and providing interoperability with established data standards such as BIDS.

To support users in adopting PyNeon, we provide comprehensive documentation available at https://ncc-brain.github.io/PyNeon/, alongside sample datasets hosted on the Open Science Framework (Chu, 2026), tutorials in the form of executable Jupyter notebooks (https://ncc-brain.github.io/PyNeon/tutorials/index.html), in addition to a contributing guide and governance statement to ensure the package’s sustainability. We welcome users to report issues, contribute code, datasets, and tutorials in various formats, including notebooks and videos. We are committed to continued support and development, ensuring that PyNeon remains a living, evolving resource – built by and for the research community.

### Outlook on enhancements and extensions

PyNeon should not be viewed as a static tool, as we expect it to be ever evolving based on the community’s needs. Here we outline two directions by which PyNeon can be further improved with the help of the community: enhancing currently implemented functionalities; and extending PyNeon to achieve novel, emerging goals.

PyNeon’s gaze mapping and scanpath estimation techniques are presently constrained by the absence of an accurate model of the visual world, making these techniques susceptible to visual disruptions. This limitation may be overcome by advanced computer vision techniques. For instance, Simultaneous Localization And Mapping [SLAM; (Abaspur Kazerouni et al., 2022)] could facilitate robust, long-term tracking even in the presence of discontinuous visual input, provide precise camera pose estimation, and enable spatial mapping. Detailed 3D reconstructions of experimental environments can also be obtained with advanced methods like Gaussian splatting (Kerbl et al., 2023). Such capabilities would provide richer insights into spatially grounded attentional behavior.

Building on PyNeon’s modular design, we envisage that the community could benefit from developing APIs that interface with diverse lines of existing research. For instance, frameworks for multi-participant eye-tracking (Saxena et al., 2025) and predictive gaze modeling (Kümmerer & Bethge, 2023) may be integrated with PyNeon’s infrastructure, further unifying methodological approaches for naturalistic behavior studies. Future extensions could include audio data processing – such as automated transcription and event detection – to enrich research in real-world settings. In this spirit, we invite conceptual and methodological contributions from the community to collectively advance the field of human neuroethology.

## Supplementary Information

Below is the link to the electronic

ESM 1 (DOCX 315 KB)

ESM 2 (MP4 44.7 MB)

## Data Availability

Sample datasets are available at 10.17605/OSF.IO/3N85H.

## Code availability

The full codebase is available at https://github.com/ncc-brain/PyNeon.

## Declarations

### Open Practices Statement

The package is open-source and hosted on GitHub at https://github.com/ncc-brain/PyNeon. The sample datasets are available at 10.17605/OSF.IO/3N85H. Tutorials for analyzing these sample datasets are available at https://ncc-brain.github.io/PyNeon/tutorials/index.html. None of the reported studies were preregistered.
