# Supplementary material for: PyNeon: A Python package for the analysis of Neon multimodal mobile eye-tracking data
Source: Behav Res Methods. 2026 Jun 29;58(8):209. doi: 10.3758/s13428-026-03089-8 (PMC13314724; doi:10.3758/s13428-026-03089-8)
Supplement: Supplementary file 1 — ESM 1 (DOCX 315 KB) [file 13428_2026_3089_MOESM1_ESM.docx]

# Supplementary Information

## Surface mapping using fiducial markers and contour

As described in the main text, one approach for mapping gaze and fixation coordinates onto surfaces relies on fiducial markers such as AprilTag or ArUco. In the *Video.detect_markers()* method, markers are first detected in video frames to establish point correspondences for surface mapping. Users can specify one or multiple marker families to detect (e.g., “36h11” for AprilTag or “5x5_100” for ArUco) and may optionally fine-tune detection parameters via the *detector_parameters* argument. These parameters include options such as adaptive thresholding settings, corner refinement strategies, and error-correction tolerance.

Internally, marker detection is implemented using the OpenCV aruco module (*cv2.aruco*). For each specified marker family, the corresponding predefined dictionary (configuration of the binary codification of each marker) is retrieved and used to instantiate an *ArucoDetector*. The *ArucoDetector.detectMarkers()* method is then applied to each grayscale video frame. This function returns the pixel coordinates of the four corners of each detected marker in the scene camera frame, together with the numeric marker identifier. Because corner ordering conventions differ between AprilTag and ArUco implementations, the detected corner coordinates are standardized to a consistent ordering: top-left, top-right, bottom-right, bottom-left. This step accounts for the differing default conventions of AprilTag detections (which start at the bottom-right corner) and ArUco detections (which start at the top-left), ensuring a unified coordinate representation across marker types.

Luminance-based contour detection provides an alternative to marker-based approaches in situations where placing physical markers on a surface is impractical or undesirable. The *Video.detect_contour()* method detects rectangular surfaces directly from image features based on brightness and geometric constraints. Users can specify several detection parameters, including brightness thresholds, area constraints, morphological kernel size, and the selection mode used to choose among candidate contours. These parameters allow the method to be tuned for different surface types and lighting conditions.

Internally, the implementation relies on standard OpenCV image-processing operations. Video frames are first converted to grayscale and may optionally be decimated to improve computational efficiency. Bright regions are isolated using either adaptive or global thresholding with a user-specified brightness value. Morphological closing is then applied to fill small gaps and smooth the resulting binary mask. Contours are extracted from the processed mask using *cv2.findContours()* with hierarchical tree retrieval and simple chain approximation. Detected contours are filtered according to their area relative to the frame size using *cv2.contourArea()*. Remaining candidates are approximated as polygons, and only those containing exactly four vertices are retained as potential surface boundaries. Corner coordinates are ordered consistently as top-left, top-right, bottom-right, bottom-left, in line with the convention used for marker detections. A geometric certainty score is computed for each candidate based on: (1) the variance of internal angles (penalizing deviation from 90°), and (2) the aspect ratio of side lengths (penalizing deviation from unity), with the combined score calculated as:

$$\mathrm{score} = -angle variance-10\times\left| aspect ratio-1 \right|$$

Depending on the specified selection mode, either the candidate with the largest area ("largest") or the candidate with the highest geometric score ("best") is returned for each frame. All relevant detection parameters—including brightness thresholds, area constraints, morphological kernel size, and selection mode—are exposed to the user to allow flexible adjustment.


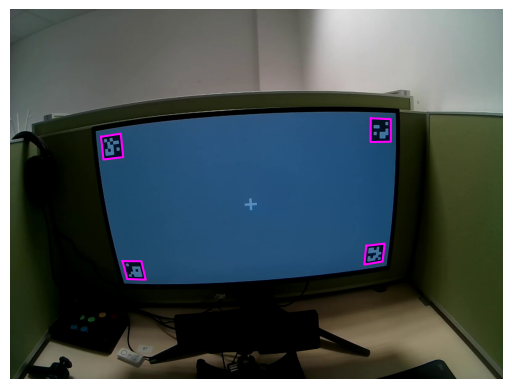

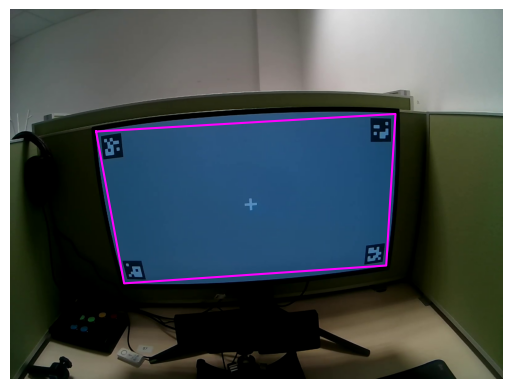


Supplementary Figure 1. Marker detection and contour detection.

Comparison of fiducial marker detection and contour detection using the same frame from the scene camera. Left: Detected ArUco markers overlaid on a frame. Right: Detected screen contour overlaid on the same frame. Both approaches can be used to establish surface mappings.

For both detection methods, optional camera undistortion can be applied to improve geometric accuracy. When enabled, each video frame is first undistorted using the camera distortion coefficients embedded in the scene video metadata. Detection is then performed on the corrected frames. To preserve the correct mapping between gaze data and the original video coordinate system, the detected coordinates are subsequently re-distorted back into the native scene camera coordinate space.

A key design challenge is enabling both marker-based and surface-based detections to be processed through a unified application programming interface (API). To address this, detection outputs from both methods are organized as *Stream* instances. Each detection record includes the frame index, timestamp, corner coordinates (top-left, top-right, bottom-right, bottom-left), center coordinates, and either the marker (e.g., “36h11_0”) or contour name (always “contour_0”). This harmonized data format ensures that detection outputs from either method can be seamlessly passed to subsequent processing steps or visualization routines (e.g., *Video.plot_detections()* or *find_homographies()*). Consequently, users can switch between marker-based and contour-based approaches or combine them without modifying downstream analysis code.

Frame-specific homographies are estimated to map coordinates from the camera video frame to a user-defined surface coordinate system. To perform this mapping, users must provide information describing the layout of the markers or surface contour in surface coordinates. For marker-based mapping, the layout is specified as a *DataFrame* describing each marker’s name, physical size, and center position within the surface coordinate system. For contour-based mapping, the user directly provides a NumPy array of shape (4, 2) containing the coordinates of the surface corners in the standard ordering described above. Users can optionally control the estimation method (least-squares, RANSAC, LMEDS, or RHO) and associated parameters, including RANSAC reprojection threshold (default 3.0 pixels), maximum iterations (default 2000), and confidence level (default 0.995). When using markers, the minimum number of detected markers required for homography estimation can also be specified via the *min_markers* argument.

In the backend implementation, corner coordinates extracted from the detection results are paired with the corresponding coordinates in the user-defined surface layout. The homography matrix (3 × 3) is then estimated using *cv2.findHomography()*, which minimizes the reprojection error between pooled scene-camera coordinates and surface coordinates according to the selected method. The resulting homography matrices are stored as a *Stream* indexed by timestamp, with each matrix flattened into its component columns. This time series of homographies enables frame-by-frame transformation of gaze coordinates or other spatial measurements from video space into surface coordinates for subsequent analysis. Because homographies are stored as flattened columns within a *Stream*, they can also be interpolated temporally beyond the original video frame rate.

Estimated homographies can be applied to gaze data (*Stream*) or fixation events (*Events*) using the *apply_homographies()* method. This operation appends two additional columns representing the transformed x and y coordinates in surface space. By default, homographies are temporally aligned with gaze or fixation timestamps by first interpolating the homography time series to the timestamps of the gaze or fixation data. As with other uses of the *interpolate()* method, users may control the maximum allowable temporal gap for interpolation using the *max_gap_ms* parameter.

We also illustrate the mapping of gaze position across scene camera and surface coordinates using ArUco markers in the Supplementary Video. The video demonstrates the gaze-mapping pipeline using a short recording in which a participant viewed a series of artworks on a computer screen. To test the robustness of the mapping, the participant was explicitly instructed to move their head during the recording. Left: Scene video with raw gaze location (red circle) and detected ArUco markers (magenta squares; numbers indicate marker IDs) overlaid during the viewing task. Right: The designated surface showing the marker layout and the corresponding mapped gaze location (red circle) in surface (pixel) coordinates. Default parameters were used throughout the mapping pipeline, except for the maximum interpolation gap when applying homographies to gaze data, which was reduced from 500 ms to 100 ms in order to adopt a more conservative interpolation criterion for this illustrative example.

## Dynamic scanpath estimation

As outlined in the main text, the scanpath tracking algorithm operates on recently completed and tracked fixations. Tracking is implemented using the Lucas-Kanade sparse optical flow algorithm *cv2.calcOpticalFlowPyrLK()*, with input video frames converted to grayscale prior to processing.

By default, the algorithm uses a three‑level Gaussian pyramid and a 90 × 90 pixel search window to accommodate large displacements. Iterative refinement terminates when positional updates fall below 0.03 pixels or after 20 iterations, whichever occurs first. All default parameters can be adjusted via the *lk_params* argument.

There are a few edge cases in which the Lucas-Kanade method can fail. First, because it relies on matching image patches between frames, it will struggle in highly uniform visual environments (e.g., a completely white room without any texture). Second, accuracy can degrade when sudden movements cause motion blur. This is an especially relevant limitation given the relatively low scene‑camera frame rate of 30 Hz.
